# Analyzing Oral Health Conditions in Sex Workers—A Comparative Retrospective Clinical and Radiographic Study

**DOI:** 10.3390/dj12040110

**Published:** 2024-04-17

**Authors:** Tahel Oguen-Alon, Leon Bilder, Hadar Zigdon Giladi, Zvi Gutmacher, Yaniv Mayer

**Affiliations:** 1 Department of Prosthodontics, School of Graduate Dentistry, Rambam Health Care Campus, Haifa 3525408, Israel; 2 Department of Periodontology, School of Graduate Dentistry, Rambam Health Care Campus, Haifa 3525408, Israel; 3 Faculty of Medicine, Technion-Israel Institute of Technology, Haifa 3200003, Israel

**Keywords:** dental caries, oral pathology, periodontitis, sex workers, women’s health

## Abstract

This study highlights the oral health condition of female sex workers (SWs), who face increased risks associated with habits such as excessive alcohol and tobacco use. These behaviors heighten the likelihood of issues like oral cancer and dental diseases, underscoring the need for targeted health interventions. The study examines the oral health disparities between SWs and the general population (GP). A retrospective study analyzed the health records of 40 SWs and 40 controls matched by age and gender who were examined between 1 January 2020 and 30 May 2023. Intra-oral and panoramic radiographs, alongside clinical examination, were used to evaluate missing teeth, periodontal bone support, and caries. *T*-tests and chi-square tests were used to compare dental health indicators. A comparative analysis of these 80 patients revealed significant disparities: SWs had a higher incidence of missing teeth (5.8 ± 7.3 vs. 0.7 ± 1.4, *p* < 0.01) and caries (6.1 ± 6.2 vs. 0.8 ± 1.2, *p* < 0.05) compared to the GP. The DMFT (Decayed, Missing, and Filled Teeth) index was notably higher in SWs (16.1 ± 8.09) than in the GP (7.95 ± 5.48, *p* < 0.001). Additionally, 12% of SWs used removable dentures, unlike the GP. This study underscores significant oral health challenges in SWs, emphasizing the need for targeted healthcare strategies to improve their health conditions.

## 1. Introduction

Oral diseases are a major global public health problem with a high prevalence and significant negative impacts on individuals, communities, and society. These diseases disproportionately affect the poorest and most marginalized groups in society and are closely linked to socioeconomic status and social determinants of health [1].

Sex work is associated with a negative social stigma which affects all aspects of sex workers’ lives, including healthcare and the attitudes of service providers and police towards them [2]. The wellbeing of sex workers is intricately linked to their social standing and the pervasive marginalization and stigmatization they face in society. This group is often subjected to criminalization, leading to unfair characterizations that paint them as deviant, disordered, or ‘vulnerable’. Such stereotypes oversimplify and distort the complex realities of sex workers’ lives [3]. These societal labels not only impact their personal dignity but also affect how they are treated within various social and legal frameworks.

Sex workers frequently endure significant mental health challenges, which are compounded by the trauma and stress associated with their working environments. These psychological burdens are often exacerbated by the precarious nature of their work, which may involve exposure to violence, societal exclusion, and constant scrutiny. The mental strain associated with managing these challenges can have profound and lasting effects on their overall health and wellbeing.

Despite facing a disproportionate health burden, female sex workers (FSWs) often struggle to access adequate healthcare and support services. This difficulty is exacerbated by high mobility rates, which can hinder consistent healthcare access and disrupt ongoing medical treatments, such as antiretroviral therapy (ART). The criminalization of sex work further complicates their ability to seek help, as it not only increases vulnerability to discrimination but also fosters an environment of fear and distrust towards healthcare providers [2].

Furthermore, concerns about stigma and discrimination from healthcare professionals can deter sex workers from accessing necessary services. These fears are sometimes compounded by language and cultural barriers, which can make communication difficult and hinder the effectiveness of the healthcare provided. The cumulative effect of these barriers is a healthcare system that often fails to meet the needs of sex workers, leaving them without the support and medical care essential for their health and survival. The exact prevalence of sex workers (SWs) is debated. It varies according to geographical location and legal status and is estimated as ranging between 0.7 and 7.4% [4]. According to a survey by the Ministry of Welfare of the State of Israel from 2016, between 11,190 and 12,040 Israelis were involved in prostitution. About 95% were women and the rest were men. Among the women, about 89% were adults and the rest were minors. Among the adult women, about 95% were women by birth and about 5% were transgender (transgender women who defined themselves as women by birth were also included under “women by birth”). Among the adult women, 93% worked in prostitution under one roof and 7% worked in street prostitution. Among women in street prostitution, about a third were transgender [5]. In addition, substance abuse such as smoking, drugs, and/or alcohol consumption was common among this population. A comprehensive cross-sectional study of female sex workers revealed that approximately 72% consume alcohol, with a significant number drinking excessively. Individuals with high alcohol consumption had significantly more teeth with decayed surfaces and apical lesions, indicating that lifestyle-related factors may influence dental health. Approximately 20% to 30% of female sex workers engage in drug and alcohol use as a coping mechanism for their profession [6]. Poor DMFT and caries and periodontal diseases among drug users may be explained by irregular tooth brushing and a long history of drug use. These behaviors, including excessive alcohol and tobacco consumption, are linked to deteriorating oral health, a condition more prevalent among at-risk groups like SWs [7]. The literature reports that SWs have a higher risk of developing oral cancer and other oral diseases due to the overlap of risk factors, including sexually transmitted infections, HPV contamination, and drug abuse, many of which are preventable, treatable, or curable [8,9]. Many studies show that this group has high rates of caries, periodontitis, and oral-mucosal lesions, but the research lacks sufficient data, uses small sample sizes, and does not compare to the general population [10,11,12]. There is a need for special attention and highlighting of the unique characteristics of this population.

The gathered data will enable an analysis of oral disease patterns and their prevalence among sex workers, informing their specific treatment requirements. This evaluation is crucial for developing a targeted treatment strategy for this community. This study seeks to compare the oral health status of sex workers with that of the general population, focusing on dental caries (DMFT index), periodontal health, and oral pathologies, both clinically and radiographically. The null hypothesis for the research stated that the oral health status, as determined through clinical and radiographic evaluation, was inferior among SWs compared to the general population.

## 2. Materials and Methods

### 2.1. Study Protocol

A retrospective study was undertaken that made use of electronic health records from Rambam Health Care Campus in Haifa, Israel. The study was approved by the Rambam Health Care Campus Ethics Committee (RMB-D-0297-23) on 14 September 2023. The study design and methodology were developed and executed by a specialist in dental public health (LB). The study group comprised 40 sex workers (SWs) involved in a social treatment project from 1 January 2020 to 30 May 2023. This group included all participants in the project. The control group consisted of 40 individuals matched with the study group in terms of age, gender, and city of residence. To qualify for the study participants had to be at least 18 years old, and to have undergone clinical examination, panoramic radiograph, and two bite-wings.

Each patient underwent a comprehensive clinical examination and panoramic radiograph and two bite-wings [13,14,15].

Panoramic radiographs were taken using the Planmeca ProMax 3D Mid machine (Planmeca OY, Helsinky, Finland) with the settings of 90 kV and 4 mA. Two bite-wing radiographs were captured using a Planmeca ProX machine (Planmeca OY, Helsinki, Finland) with the settings adjusted to 60 kV and 8 mA, utilizing a Planmeca ProSensor (Planmeca OY, Helsinki, Finland).

### 2.2. Outcome Variables

All data were extracted from patient records and evaluated by one author (LB) with further validation by the primary author. Dental caries assessments were conducted using bite-wing radiographs and clinical examinations. All clinical parameters were assessed by the same examiner, who was both blinded and calibrated (T.O.A).

**Figure 1:**
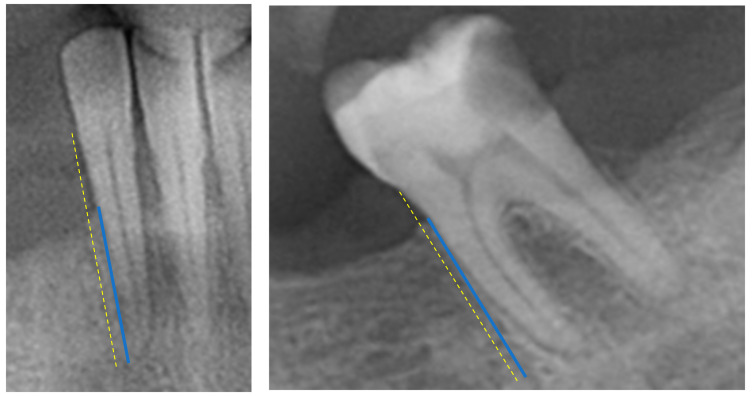
Radiograph-based Periodontal Bone Loss (R-PBL) was measured from the marginal alveolar bone to the tooth apex (blue line) and from the cementoenamel junction to the tooth apex (yellow dotted line).

- The DMFT index (Decayed, Missing, and Filled Teeth index) was calculated by adding together the number of teeth that were decayed (D), missing (M), or filled (F) due to caries (tooth decay). Each tooth was examined and categorized into one of these three conditions, and the total count gave the DMFT score [13].
- Periodontal Bone Loss (PBL)—the radiographic PBL was assessed by measuring the total root length (distance from the tooth’s apex to the cementoenamel junction) and the total bone height (distance from the tooth’s apex to the marginal alveolar bone) in each tooth, as described in previous research [14] (Figure 1). Measurements were performed with a high-resolution computer monitor in a darkened room. For these measurements, the arithmetic mean was then calculated and used as a measure of proportion (%). Based on the PBL, as a percentage, patients were then divided into different groups: Healthy Periodontium (if PBL ≥ 80%), Mild-to-Moderate Periodontitis (if PBL ranged between 79 and 66%), and Severe Periodontitis (if PBL < 66%) [15]. These measurements were conducted using ImageJ software (Image Tool 3.0 software program, Department of Dental Diagnostics Science, University of Texas Health Science Center, San Antonio, TX, USA).
- Dental anomalies and oral pathologies—Periapical diseases and other findings. Detection of dental diseases such as periapical lesions, impacted or missing teeth, maxillary sinus anomalies, and condylar changes and other miscellaneous findings.
- Prosthetics—The number of crowns and implants was calculated for each patient.
- Demographic data such as age, sex, and medical status were taken from the electronic data.

### 2.3. Intra-Observer Reproducibility

In this study, we conducted a comprehensive evaluation of the intra-observer reproducibility of our measurements to ensure their reliability and accuracy. The observer (TOA) carried out ten repeated measurements, which were then meticulously analyzed to detect any inconsistencies or variations. We employed point estimates and a 95% Confidence Interval (CI) for assessing the differences in these measurements. Intra-Class Correlation Coefficient (ICC) scores for Periodontal Bone Loss measurements were high at 0.92 and 0.90. This indicates a high level of reproducibility.

### 2.4. Sample Size

Due to the lack of available data needed to determine the effect size, a pilot study approach was selected. The study included a total of 80 participants, comprising 40 patients who were sex workers (encompassing all individuals participating in the governmental project) and 40 control patients.

### 2.5. Statistical Analysis

The statistical analysis for this study was conducted using IBM SPSS Statistics software, version 25.0. Given the non-normal distribution of our data, nonparametric Mann–Whitney U tests were applied for bivariate analyses to compare the Decayed, Missing, and Filled Teeth (DMFT) index across the variables of age and sex. Descriptive statistics, including means and standard deviations, were calculated to summarize the dataset. A *p*-value threshold of less than 0.05 was set for determining statistical significance.

## 3. Results

For each group, 40 electronic health records were scrutinized, with the findings summarized in Table 1. The average age among the sex worker group was 41.8 ± 12.29 years, which was not statistically different from the control group’s mean age of 37.31 ± 12.88 years. Within the sex worker cohort, 70% (28 individuals) identified as female, whereas the control group comprised 57.5% (23 individuals) female participants.

**Figure 2:**
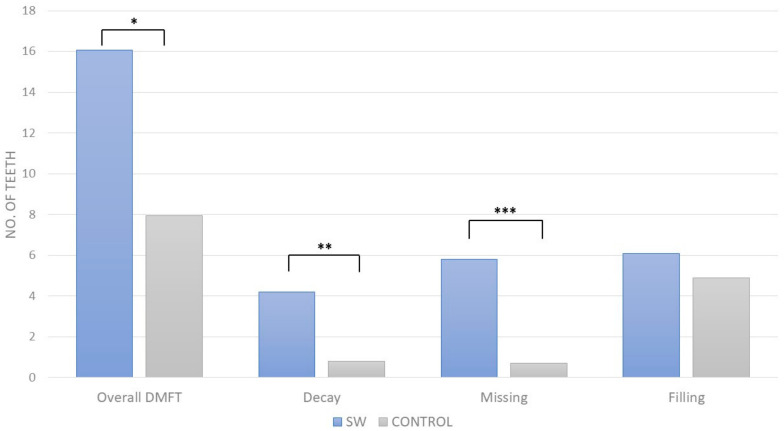
DMFT index in sex worker (SW) group and control group; *—*p* < 0.001, **—*p* < 0.01, ***—*p* < 0.05.

The sex worker group exhibited a notably higher mean DMFT (16.05 ± 8.09 vs. 7.95 ± 5.48, *p* < 0.001), indicating severe decay, missing, and filled teeth conditions. Figure 2 demonstrates the overall DMFT index and its separate parameters. Additionally, the prevalence of peri-apical lesions was significantly higher among sex workers (1.95 ± 2.22 vs. 0.5 ± 0.98, *p* < 0.001), and they were also more likely to have decayed teeth (4.2 ± 4.1 vs. 0.8 ± 1.2, *p* < 0.05) and supragingival calculus (60% vs. 17.5%, *p* < 0.001). Missing teeth were more common in the sex worker group (5.8 ± 7.3 vs. 0.7 ± 1.4, *p* < 0.01).

In both study groups, a regression analysis conducted to examine the relationship between age and the number of missing teeth, as well as between age and the DMFT (Decayed, Missing, Filled Teeth) score, indicated a moderate correlation. The correlation coefficient (r) was found to be less than 0.5 for both parameters in each group (data not shown).

Within the SW group, there was no difference between males and females (*p*-value > 0.05). Conversely, in the control group, a marginally significant variance was noted in the DMFT score between males and females (*p* = 0.053), alongside a statistically significant difference in the amount of teeth with fillings (*p* = 0.02), as detailed in Table 2. 

Within the sex worker group, one patient experienced an oro-antral fistula following the extraction of a molar tooth, while another patient from the same group was diagnosed with nasal cavity candidiasis. There were no mentions of soft tissue lesions in the control group, according to our records.

These results suggest that the SW group generally had poorer dental health compared to the Control group, with significant differences in DMFT, peri-apical lesions, decayed teeth, supragingival calculus, and missing teeth. However, there were no significant differences in periodontal bone loss, wisdom teeth, restorations, root canal treatments, or dental implants between the two groups. 

## 4. Discussion

This study focused on the oral health disparities between female sex workers (SWs) and the general population (GP). The retrospective study analyzed health records from 80 individuals (40 SWs and 40 matched controls) using clinical examinations and radiographic imaging to assess dental health indicators like the presence of caries, missing teeth, and periodontal bone support. Significant findings include a higher incidence of caries and missing teeth among SWs compared to the GP, underscoring the need for targeted health interventions to address these disparities. 

The Prohibiting of Consumption of Prostitution law of 2019 came into force on July 10, 2020. The law imposes a fine for accessing acts of prostitution and for attempting to access prostitution. With the adoption of the law, Israel became the eighth country in the world to prohibit the accessing of prostitution. In 2019, after the law came into force, the Israeli Ministry of Health created a special program for the oral health rehabilitation of SWs. The School of Graduate Dentistry, Rambam Health Care Campus (Haifa, Israel), was chosen as the provider of the program and treated the SW population during 2020–2023. This was the first time that a study had been conducted in Israel about oral health in the population of prostitution workers.

The DMFT index, which assesses Decayed, Missing, and Filled Teeth, shows a marked disparity between the group of sex workers (SW) and the control group (*p* < 0.001), highlighting a notably higher occurrence of dental issues in sex workers than in the control group. 

Minor variations in periodontal bone loss and marginal bone height were observed between the groups, but these differences were not statistically significant. This indicates that dental decay and neglect are more pressing issues for the sex worker (SW) group than periodontal disease, likely due to their relatively younger age, as periodontal conditions typically develop later in life. The SW group exhibited significantly higher mean values for periapical lesions, decayed teeth, and supragingival calculus (*p* < 0.001 for lesions and calculus; *p* < 0.05 for decayed teeth), which points to more severe dental pathologies among sex workers. Additionally, this group had a notably higher average number of missing teeth (*p* < 0.01), suggesting a higher incidence of tooth loss, likely due to untreated caries and poor oral hygiene. No significant differences were found in the average number of fillings and root canal treatments between the groups, possibly because sex workers may not seek treatment for caries as frequently, leading to fewer dental restorations. Overall, these findings highlight a distinct disparity in oral health outcomes between sex workers and the control group, characterized by higher rates of dental caries, tooth loss, and untreated caries rather than advanced periodontal disease. Further research is needed to identify specific contributing factors and to develop interventions to improve the oral health of sex workers.

Compared with clinical examination, radiographs are very helpful for detecting dental pathologies such as periapical lesions, impacted or missing teeth, maxillary sinus anomalies, and condylar changes, which cannot be seen in clinical examinations. A panoramic radiograph was the most effective method for detecting impacted teeth and other miscellaneous findings due to the greater area of coverage. With a panoramic examination, it is possible to detect periapical lesions and other abnormalities which cannot be detected in clinical examination [16,17]. Several studies have demonstrated the advantage of panoramic radiography for the diagnosis of periodontal disease. Choi reported 62.6% calculi deposition in screening panoramic radiographs, which was higher than that found by clinical examination by 7.4%. Moreover, he reported that panoramic radiographs detected more periodontal bone loss than periapical radiographs [16]. These findings indicate that panoramic radiography is a useful method in the diagnosis of periodontal disease and can improve the detection of periodontal diseases.

Bite-wing X-rays were used to identify interproximal caries, while occlusal caries were documented in each patient’s electronic records. All X-rays were captured using the same machine with identical settings and reviewed by a single calibrated dental surgeon. Relevant data were gathered from panoramic and bite-wing X-rays, and in certain instances additional periapical X-rays were utilized.

A comparison with the findings in previous studies provides valuable insights into the oral health status of female sex workers and underscores the challenges they face in maintaining optimal oral health. Different studies have highlighted the prevalence of caries, periodontitis, and oral mucosal lesions in this population [7,9,10,11]. However, few studies have compared the oral health status of sex workers to that of the general population using radiographic assessments.

In our study, the DMFT score was higher compared to findings in previous studies [10,12]; the same is true for dental calculus [18]. We did not find similar articles that examined the population of SWs and which analyzed the parameters we have examined in our research.

Our findings in the control group reveal that females exhibited a higher number of teeth with fillings compared to males. This observation aligns with existing literature that suggests females are more likely to visit dental clinics regularly. For instance, a study by Furuta et al. supports the notion that women tend to seek dental care more frequently than men, potentially contributing to the higher incidence of filled teeth observed in our female participants [19].

This lack of disparity could be attributed to the smaller sample size of males within this subgroup or the possibility that individuals in this group do not regularly attend dental clinics, negating the gender-based differences in dental care utilization observed in the general population. 

This research addresses an important knowledge gap, delivering comparative insights into the distinctive patterns of oral and periodontal status among sex workers. It implies that the specific social and professional situations of sex workers could impact their approaches to seeking healthcare, highlighting the necessity for specialized health interventions and studies aimed at mitigating these differences.

There are several limitations in this study that are related mainly to the nature of the SW population. The study was conducted with a small sample size, which may limit the generalizability of the findings to the broader population of female sex workers. The study was conducted in a single center without comparison to other centers, which limits the ability to assess variations in oral health status across different settings or regions. Another concern is that there might be a selection bias in the study population, as not all female sex workers may have been willing to participate in the program. It is possible that sex workers whose dental condition is more challenging refused to participate the program. This potential disparity could affect the results of this study and limit our ability to understand the severity of the dental condition in the SW population. Furthermore, the broad age range and absence of prior records prevented us from precisely determining whether missing teeth are due to caries or periodontal disease. It is important to acknowledge that the control group was not matched with the study group in terms of socioeconomic status, as reflected by education level, nor were they matched for diet and oral care habits at home. These factors could significantly influence the pronounced discrepancies observed between the groups, potentially skewing the results and affecting the interpretation of the oral health disparities highlighted in this study. It is important to note that, according to the 2016 National Survey on Prostitution in Israel, the number of individuals engaged in prostitution was estimated to be between 11,190 and 12,040. Therefore, our group of sex workers (40) constituted about 0.15% of this total SW population. 

When reflecting on these findings and planning future research, addressing the limitations identified in this study is crucial. Future research should explore the oral health needs of female sex workers more thoroughly by not only assessing their oral health status, but also examining their knowledge and practices regarding oral care. This expanded approach will offer a more comprehensive understanding of their needs and facilitate the development of targeted interventions aimed at enhancing their oral health outcomes. This effort is essential to devise strategies that effectively improve their wellbeing and address the specific challenges they face in maintaining oral health. By integrating a broader perspective that includes behavioral insights and access issues, researchers can design more effective and sustainable health promotion programs tailored to this underserved population [20].

## 5. Conclusions

This study highlights the substantial oral health disparities among sex workers, underscoring the need for specialized treatment and preventive care strategies to address these challenges and improve overall wellbeing within this marginalized group. Considering all the limitations mentioned, these findings emphasize the urgency of tailored interventions that can effectively reach and benefit this vulnerable population. To improve dental care for female sex workers, it is crucial for dental students and practicing dentists to undergo sensitivity training. This training should enhance their understanding of sex work, address the unique dental needs of sex workers, and equip them with effective communication techniques. A deep appreciation of the challenges sex workers face will allow dentists to empathize and establish a meaningful rapport with these patients. Furthermore, dentists should be encouraged to engage in broader discussions about the health needs of sex workers and to share their clinical experiences with colleagues. This exchange of knowledge will raise awareness about the specific health issues this group encounters and explore effective strategies to enhance their dental care.

## Figures and Tables

**Table 1:** Demographic data and clinical parameters in the sex worker (SW) group and control group.

	SW Group (*n* = 40)	Control Group (*n* = 40)	
Age, mean ± SD (max, min)	41.8 ± 12.29 (79, 28)	37.31 ± 12.88 (74, 21)	NS
Gender (female/male)	28/12	23/17	NS
DMFT, mean ± SD	16.05 ± 8.09	7.95 ± 5.48	*p* < 0.001
**Periodontal pathologies**			
Periodontal Bone Loss, mean ± SD	1.42 ± 0.63	1.27 ± 0.50	NS
General marginal bone high (% of root length)			
>80%	9 (22.5%)	15 (37.5%)	NS
≥66% but <79%	16 (40%)	15 (37.5%)	NS
<65%	15 (37.5%)	10 (25%)	NS
**Dental pathologies**			
Peri-apical lesion, mean ± SD	1.95 ± 2.22	0.5 ± 0.98	*p* < 0.001
Decayed teeth, mean ± SD	4.2 ± 4.1	0.8 ± 1.2	*p* < 0.05
Supragingival calculus (% patients)	60%	17.5%	*p* < 0.001
Wisdom teeth (impacted/unerupted), mean ± SD	1.52 ± 5.4	2.15 ± 5.76	NS
Missing teeth, mean ± SD	5.8 ± 7.3	0.7 ± 1.4	*p* < 0.01
**Restorations**			
Filling teeth, mean ± SD	6.1 ± 6.2	4.9 ± 3.4	NS
Root canal treatments, mean ± SD	3.1 ± 5.5	1.4 ± 2.4	NS
Removable prosthesis (% patients)	12%	0%	---
Dental implants (no. implants/no. patients)	15/4	15/8	NS

NS—Non-significant.

**Table 2:** Difference in DMFT and periodontal bone loss between females and males in SW group and control group.

	SW Group (*n* = 40)	Control Group (*n* = 40)
DMFT	*p* = 0.657	*p* = 0.05
Decayed teeth	*p* = 0.511	*p* = 0.489
Filling teeth	*p* = 0.956	*p* = 0.02
Missing teeth	*p* = 0.167	*p* = 0.530
Periodontal bone loss	*p* = 0.906	*p* = 0.588

## Data Availability

Data are contained within the article.
